# Evidence for the Circulation of Equine Encephalosis Virus in Israel since 2001

**DOI:** 10.1371/journal.pone.0070532

**Published:** 2013-08-12

**Authors:** David G. Wescott, Zvia Mildenberg, Michel Bellaiche, Sarah L. McGowan, Sylvia S. Grierson, Bhudipa Choudhury, Falko Steinbach

**Affiliations:** 1 Department of Virology, Animal Health and Veterinary Laboratories Agency, Weybridge, Surrey, United Kingdom; 2 Kimron Veterinary Institute, Bet Dagan, Israel; Blood Systems Research Institute, United States of America

## Abstract

Equine encephalosis virus (EEV) distribution was thought to be limited to southern Africa until 2008 when we reported EEV in Israel. It was then assumed that the clinical presentation resembled the initial incursion in Israel. To investigate further we conducted a retrospective analysis of equine sera, which had been collected for diagnosis of other suspected diseases, via serum neutralisation test. The data demonstrated that EEV was circulating as early as 2001 with incidence ranging from 20–100% for time period 2001–2008. As the symptoms of EEV can be similar to other equine notifiable diseases this is a significant finding which highlights the need for vigilance and education to accurately diagnose new and emerging diseases.

## Introduction

Equine encephalosis virus (EEV) is an Orbivirus, family *Reoviridae*. The genome consists of 10 double stranded RNA segments of which the second segment (VP2) can used to determine serotype [1] To date; seven antigenically distinct serotypes of EEV have been identified [2]. EEV is an acute arthropod-borne viral infection that infects all species of equids and is transmitted by certain *Culicoides* species. It was first isolated in South Africa in 1967 and is thought to be endemic in this region. Until 2008 and its appearance in Israel [3], EEV distribution was thought to be limited to southern Africa. Recently, a serological study reported the virus to be endemic in countries of East and West Africa [4].

Some of the symptoms observed in cases of EEV are common to cases of other notifiable diseases, particularly African Horse Sickness (AHS) and Equine Viral Arteritis (EVA) making EEV important as a differential diagnosis [2]. We reported an undiagnosed disease in several horses in four stables in the Hamerkaz region in central Israel from an outbreak which started in October 2008 [3]. Symptoms included fever (up to 41°C), depression, anorexia, edema, muscle pain, stiffness, generalised weakness, cough and in some cases nasal discharge and conjuctivitis. In the following three weeks the disease spread throughout the country with 42 recorded outbreaks and an estimated 80% of the country's horse population effected. The disease affected all breeds, ages and sexes. Ninety percent of horses recovered from the disease without additional complications and no deaths were reported. Initially, the disease was thought to be EVA but serological tests for this, influenza, equine rhinopneumonitis and West Nile virus all gave negative results. Additional tests for toga and flavi viruses were also negative. The causative agent was finally identified as EEV following DNA array analysis, with subsequent RT-PCR and sequence analysis [3]. As EEV disease symptoms could be easily confused with that of other viruses it was of interest to investigate whether this was truly the first incursion or whether EEV had been circulating previously in Israel. To undertake this objective we conducted retrospective serological analysis of equine serum samples from Israel dating back to 2001. In addition we undertook full genome sequencing of an isolate obtained from the beginning of the 2008–9 outbreak in order to gain a deeper understanding with regards to origin of the Israeli outbreak.

## Materials and Methods

### Sample selection for retrospective serological analysis

Samples were selected from archived material which was submitted to the Kimron Veterinary Institute during the period 2001–2008 for diagnosis of other suspected diseases, but for which a laboratory diagnosis was not reached. The samples were selected on basis that clinical examination suggested symptoms associated with notifiable diseases. The investigated samples were from foals (9.2%), geldings (45.4%), mares (42.9%) and stallions (2.5%). Over 50% of the samples were from horses housed along the Mediterranean Coast with the remainder originating from various in-land regions across northern Israel. Symptoms observed in the sample set included respiratory, neurological and reproductive (abortions). None of the sampled animals had travelled outside of Israel. Sample collection was conducted according to the animal welfare committee guidelines of the Kimron Veterinary Institute, which also approved the use of the samples for this study.

### EEV Virus Isolation

1 ml of blood was inoculated onto 1 day old 25 cm^2^ flask of Vero cells (ATCC) at 90% confluent monolayers. The flasks were incubated for 1 hour at 37°C in 5% CO_2_ to allow the virus to adsorb and then the inoculums washed off and overlaid with 10 ml of MEM supplemented with 5% fetal bovine serum (Life Technologies, UK). The cultures were examined daily for up to 5 days incubation, before being harvested by freeze/thawing and passaging onto fresh cell cultures.

### Virus neutralisation assay

The virus neutralisation assay was essentially that described by Senne *et al.* [5], for EVA except that rabbit complement was not was included in the addition of EEV. To conduct serological analysis, a virus isolate from the beginning of the 2008–9 outbreak, Kimron1, was cultured and titred to determine the TCID_50_ per ml. Serial twofold dilutions of the test sera (heat treated at 56°C for 30 minutes for complement inactivation) were made in duplicate in serum free cell-culture medium using a 96-well microtitre plate starting at a 1∶2 serum dilution. Using serum-free cell culture medium as a diluent, a dilution of stock virus was prepared containing 100 TCID_50_/ml. A virus back titration of the working dilution of stock virus was included with each test. The plates were incubated for 1 hour at 37°C in a humid atmosphere of 5% CO_2_ in air. A suspension of Vero cells was added and the plates were returned to the incubator for 3–5 days before reading for cytopathic effect. To obtain a more accurate reading of the titre end-points an immunoperoxidase stain was performed as described by Smith *et al.*, [6]. End points were calculated using the Spearman-Kärber method [7].

### Full genome sequencing and analysis

The Kimron1 isolate was used as the starting material for next generation sequencing via the Roche 454 FLX platform following protocols described by Ramussen *et al.*, 2008 and 2010 [8]–[9]. Additional sequences were obtained from GenBank for comparison. Nucleotide alignments were conducted using Clustal W [10].

## Results and Discussion

The serological analysis revealed antibody positives in each year analysed back to 2001 (Table 1). The percentage varied between 20–100%, however these data should only be seen as demonstrating previous infection as this study was not designed as an epidemiological survey.

**Table 1:** Serological analysis for EEV antibodies in Israeli equine sera 2001–2008.

Sample date (Year)	Number of samples tested	Number of positives	Percentage positive
2001	5	4	80
2002	5	1	20
2003	5	5	100
2004	5	2	40
2005	5	1	20
2006	10	3	30
2007	8	3	37.5
2008	76	57	75

The resultant full genome sequence of Kimron1 (accession: AB811630-9) was compared to other publicly available EEV sequences. Here, we were limited as full genome data is only available for one other EEV strain HS103/06 Bryanston (accession FJ183384-93). In comparison to HS103/06 the greatest diversity was observed in comparison of the VP2 gene (54% at the nucleotide level) and greatest similarity was observed in comparison of the VP7 gene (96% at the nucleotide level). Analysis of other VP2 sequences revealed closest similarities to Kaalpaas serotype 3 strain (92% at a nucleotide level), Israeli isolates from 2008–9 outbreak described by Aahronson-Raz *et al.* [11], (99.8% homology) and a Gambian isolate from 2009 described by Oura *et al.* [4], (99% nucleotide homology) (accession: HQ630933, JF495411, JF495412 and JN391443 respectively). Analysis of the NS3 revealed 99.9% homology with a 2009 Israeli isolate [11] (accession: HQ441245). Further investigation into genetic origin was not possible due to lack of sequence data for comparison. We attempted genomic amplification on the samples used in the retrospective serological analysis; this was unsuccessful with alternate samples from the same animals not being available.

The similarity in the clinical presentation of EEV with other notifiable diseases, particularly EVA, is demonstrated by the symptoms and history of the 2008–9 outbreak. The detection of EEV outside of Africa highlights the risk of its spread to other regions where susceptible animals and suitable vectors are present, it is thus essential to understand the distribution of EEV. Here we demonstrate that a very similar strain of EEV, belonging to the same serotype, was already circulating in the Mediterranean region during a time period in which it was thought that the virus circulation was limited to southern Africa. None of the animals investigated had travelled outside of Israel therefore infection must have been acquired in country. From 2001 onwards at least, EEV infection was either overlooked or mis-diagnosed: demonstrating both the importance of vigilance and education to diagnose new and emerging diseases with the assistance of laboratories equipped to carry out investigative virology.

Full genome sequence data will be of interest to those wishing to undertake further investigation of this disease. As EEV has a segmented genome it would be of interest to investigate whether recombination events contributed to the virulent strain observed in the 2008–9 Israeli outbreak. The similarity of the Israeli VP2 sequences to the 2009 Gambian isolate [4] may provide inference regarding the origin of the Israeli outbreak however thus far a lack of sequence data prevents further analysis, highlighting the need of additional sequencing if EEV is to be accurately analysed in its temporal and spatial distribution.
