# Correction: Evidence for the Circulation of Equine Encephalosis Virus in Israel since 2001

**DOI:** 10.1371/annotation/4875ab92-466a-4f5f-b9c7-bc0e168a8f9b

**Published:** 2013-09-30

**Authors:** David G. Westcott, Zvia Mildenberg, Michel Bellaiche, Sarah L. McGowan, Sylvia S. Grierson, Bhudipa Choudhury, Falko Steinbach

There was an error in the name of the first author. The correct name is: David G. Westcott.

The correct citation is: Westcott DG, Mildenberg Z, Bellaiche M, McGowan SL, Grierson SS, et al. (2013) Evidence for the Circulation of Equine Encephalosis Virus in Israel since 2001. PLoS ONE 8(8): e70532. doi:10.1371/journal.pone.0070532 

